# Lipid Exchange Factors at Membrane Contact Sites in African Swine Fever Virus Infection

**DOI:** 10.3390/v11030199

**Published:** 2019-02-26

**Authors:** Inmaculada Galindo, Miguel Ángel Cuesta-Geijo, Ana del Puerto, Eva Soriano, Covadonga Alonso

**Affiliations:** Department Biotecnología, Instituto Nacional de Investigación y Tecnología Agraria y Alimentaria (INIA), Ctra. de la Coruña km 7.5, 28040 Madrid, Spain; galindo@inia.es (I.G.); cuesta.miguelangel@inia.es (M.Á.C.-G.); delpuerto.ana@inia.es (A.d.P.); e.sorianojerez@pgr.reading.ac.uk (E.S.)

**Keywords:** lipids, viral factory, itraconazole, 25-HC, OSBP, PI4Kβ, ACBD3, membrane contact sites, sterol transport, African swine fever virus

## Abstract

African swine fever (ASF) is a hemorrhagic fever of wild and domestic pigs with a high rate of mortality. Originally endemic in Africa, this disease is currently disseminating in Europe and China, causing a large socioeconomic impact. ASF is caused by a DNA virus, African swine fever virus (ASFV). There is no vaccine available against ASFV, limiting the options for disease control. ASFV reorganizes intracellular membranes to generate viral factories (VFs) in order to amplify its genome. However, little is known about the process involved in the formation of these viral replication organelles. Membrane contact sites (MCSs) allow nonvesicular lipids and ion exchange between organelles. Lipid exchange to form VFs apparently requires a number of proteins at MCSs, such as the oxysterol-binding protein (OSBP), the acyl-coenzyme A binding domain containing 3 (ACBD3) and the phosphatidylinositol-phosphate-4-kinase III beta (PI4Kβ). Itraconazole (ITZ) is an antifungal agent that targets sterol-transport molecules such as OSBP and OSBP-related protein 4 (ORP4). 25-Hydroxycholesterol (25-HC) inhibits lipid transport by high affinity binding OSBP. In this work, we analyzed the antiviral function of ITZ and 25-HC against ASFV in Vero cell cultures using the cell-adapted Ba71V isolate. ITZ and 25-HC decreased significantly ASFV replication. Our study revealed OSBP distribution in cytoplasmic membranes in uninfected Vero cells and to the periphery of VFs in infected cells. In addition, we showed that OSBP and OSBP-related proteins, PI4Kβ and ACBD3 were recruited to VFs in the context ASFV infection.

## 1. Introduction

African swine fever virus (ASFV) causes a highly contagious disease affecting wild and domestic pigs. African Swine Fever (ASF) causes severe economic losses for pig industry due to the high rate of mortality associated with the illness and the difficulties to control the disease in the absence of an effective vaccine [1,2]. Currently, a large epizootic outbreak is affecting eastern and western Europe. The latest reports of the disease include an increasing list of affected provinces in China [3].

ASFV is the only member of the Asfarviridae family [1]. It is an enveloped virus with icosahedral morphology and an average diameter of 200 nm. The genome structure consists of a linear double stranded DNA with terminal inverted repeats and hairpin loops [4]. The ASFV genome is 170–190 kbp length and encodes between 151 and 167 open reading frames (ORFs) [5,6,7]. Its replication cycle is mainly cytoplasmic. However, it requires the nucleus for viral DNA synthesis at early times [8,9].

ASFV infectious cycle starts with viral adsorption and entry into the host cell by binding an unknown specific receptor that mediates endocytosis [10]. Viral entry is a complex process that progress into the endocytic pathway [11,12,13]. Successful viral infection requires cholesterol pathway integrity at several levels in the target cell [14]. ASFV uncoating depends on decapsidation at low pH of endosomes [15]. Furthermore, cholesterol transport is necessary for fusion between the viral membrane and the endosome to deliver DNA into cytoplasm in order to start replication. Blocking cholesterol transport at this level causes retention of virions inside endosomes, inhibiting infection progression [14]. Moreover, several molecules related to endosomal signaling are also required for replication, as shown for Rab 7 GTPase and rare lipids called phosphoinositides (PIs) at the endosomal membrane [15].

Once viral DNA has been released to the cytoplasm, it accumulates into a perinuclear area close to the microtubule-organizing center (MTOC) [16]. Replication and assembly of new virions takes place in the replication sites or “viral factories”. New progeny virions leave the factory and are transported to cell surface where they get released by budding [17]. Similar to other viruses, in order to amplify their genomes, ASFV reorganizes intracellular membranes to generate replication organelles (ROs) or viral factories (VFs) in the cytoplasm [18]. Despite their relevance for virus replication, the mechanisms and molecules underlying VFs formation are still unknown. Recent scientific evidence demonstrated that lipids are fundamental for the formation of VFs in several viral infections. Diverse viruses use membrane contact sites (MCSs) to ensure lipid transfer to VFs [19,20]. Resident molecules at MCSs allow the exchange of non-vesicular lipids and ions between organelles [21]. Signaling lipids regulate MCSs formation providing energy to transfer other lipids, such as phosphatidylinositol-4-phosphate (PI4P). PI4P is locally produced at mammalian endoplasmic reticulum (ER)-Golgi MCSs by phosphatidylinositol-phosphate-4-kinase III beta (with several isoenzymes such as PI4KIIIb or PI4Kβ). PI4P is responsible of the docking of oxysterol-binding protein (OSBP), a lipid-binding/transfer protein, to the Golgi. OSBP transports cholesterol from the ER to the Golgi, where cholesterol accumulates, followed by back transfer of PI4P. PI4P hydrolysis at the ER provides energy to this process [21].

Several studies have implicated the MCSs machinery and PI4P-dependent sterol shuttling proteins in viral replication. Viruses would recruit a host PI4P kinase to enrich PI4P at their replication organelles. In all cases, PI4P lipids serve to recruit OSBP to the viral factories, leading to the formation of MCSs between the ER and the VFs at which a cholesterol/PI4P exchange drives the accumulation of cholesterol at the site of replication [21]. On the other hand, the Golgi adaptor protein acyl-coenzyme A binding domain containing 3 (ACBD3) is required to recruit PI4Kβ to VFs in a number of RNA viruses [22,23]. In uninfected cells, PI4Ks must be recruited to the correct membrane compartment to convert their lipid substrates. More specifically, ACBD3 is capable of recruiting PI4Kβ to membranes that are critical for the maintenance of the Golgi PI4P pools. This recruitment increases its enzymatic activity. The ACBD3:PI4KB complex formation is essential for a proper function of the Golgi [24]. Also, this system could be targeted by antivirals. ITZ is a triazole antifungal agent that inhibits cytochrome P-450-dependent enzymes required for sterol synthesis [25] and sterol shuttling at cellular membranes [26]. ITZ has been found to have anticancer activity and to downregulate mTOR signaling [27], consequence of impeded intracellular cholesterol transport [28]. Also, 25-HC inhibits lipid transport by high-affinity binding OSBP [29,30].

Then, our goal was to study the effect of ITZ and 25-HC on ASFV infection in Vero cell cultures using the cell-adapted Ba71V isolate and the possible interaction of ASFV with intracellular lipids and proteins at MCSs that have been related to lipids exchange in order to form the VFs.

## 2. Materials and Methods

### 2.1. Cells, Virus and Infections

Vero and HeLa cells, from American Type Culture Collection (ATCC), were grown at 37 °C, 5% CO_2_ atmosphere in Dulbecco’s Modified Eagle’s Medium (DMEM), supplemented with 5% fetal bovine serum (FBS), or 2% for viral infections, containing penicillin/streptomycin (P/S) and Glutamax (G; Gibco, Gaithersburg, MD, USA). Ba71V is the cell culture-adapted non-pathogenic ASFV isolate [31] and BPP30GFP is a recombinant African swine fever virus, expressing GFP gene fused to the promoter of the early viral p30 protein [32]. Viruses were partially purified by a sucrose cushion (40%) in PBS at 49,400 *g* for 50 min at 4 °C and were used at a multiplicity of infection (moi) of 1 pfu/cell. When synchronization of infection was required, virus adsorption was performed for 90 min at 4 °C followed by a phosphate-buffered saline (PBS) wash to remove unbound virus and then, cells were rapidly shifted to 37 °C with fresh medium.

### 2.2. Virus Titration

Vero cells were infected with ASFV at a moi of 1 pfu/mL after drug treatment. Total virus was collected at 24 postinfection and titrated by plaque assay on monolayers of Vero cells. Cells were inoculated with 10-fold serial dilutions from samples for 90 min at 37 °C. The inoculum was removed and semisolid medium added (1:1 low melting-point agarose and 2× minimal essential medium [MEM]). Plaques visualization was possible at 10 days after staining with violet crystal.

### 2.3. Reagents

ITZ was purchased from Santa Cruz Biotechnology and 25-Hydroxycholesterol (25-HC) from Sigma Aldrich (St. Louis, MO, USA). Rabbit polyclonal anti-OSBP (Sigma) was used at 1:50 dilution, anti-PI4Kβ (Millipore, Burlington, MA, USA) at 1:300 dilution, anti-ACBD3 (Sigma-Aldrich) at 1:100 dilution and mouse monoclonal antibody to PDI (ab2792 abcam) at 1:1000; MVB were labeled with anti-CD63 antibody (Clone H5C6; Developmental Studies Hybridoma Bank, University of Iowa) at 1:200 dilution. MitoTracker CMXRos was obtained from Invitrogen (Waltham, MA, USA) and used at 100 nM. Other primary antibodies used were monoclonal antibodies against the virus major capsid protein p72 (Ingenasa, Madrid, Spain) used at a working dilution of 1:1000, anti-p30 antibody at 1:100 dilution (kindly given by Dr. J.M. Escribano, INIA) and swine anti-p54 antibody at 1:100. As secondary antibodies, we used anti-mouse immunoglobulin G (IgG) antibody conjugated to Alexa Fluor 594 and anti-rabbit IgG antibody conjugated to Alexa Fluor 488 (Molecular Probes, Eugene, OR, USA), both at 1:200 dilutions and FITC-conjugated anti-mouse inmmunoglubulins 1:50, diluted in FACS buffer (Dako, Agilent, Santa Clara, CA, USA).

### 2.4. Drugs Treatments

Vero cells were pretreated for 1 h at 37 °C with ITZ, 25-HC or DMSO solvent at the indicated concentrations followed by cold synchronized infections. Stock solutions were prepared in DMSO or ethanol respectively and dissolved in cell culture medium to give a final concentration.

### 2.5. Cytotoxicity Assays

Vero cells seeded in 96-well plates were incubated with DMEM containing ITZ or 25-HC at concentrations ranging from 0 to 200 μM. After 24 h incubation, the cytotoxicity was analysed using the CellTiter 96 AQueous Non-Radiactive Cell Proliferation Assay (Promega, Madison, WI, USA) following the manufacturer’s instructions based in the reduction to formazan of 3-(4,5-dimethylthiazol-2-yl)-5-(3-carboxymethoxyphenyl)-2-(4-sulfophenyl)-2H-tetrazolium (MTS). Then, we selected optimal nontoxic working concentrations to test the activities of these compounds on ASFV infection.

### 2.6. Detection and Quantification of Viral DNA

The quantitation of the number of copies of ASFV genome was achieved by quantitative real-time PCR (qPCR). DNA was purified from Vero cells treated with the indicated concentration of ITZ and infected with ASFV at a moi of 1 pfu/cell for 16 h post infection (hpi), using the kit “Dneasy blood and tissue” (Qiagen, Hilden, Germany) following the Manufacturer’s instructions. DNA concentration was measured with Nanodrop. Uninfected cells and cells infected in the absence of drug were used as controls. The PCR assay used fluorescent hybridization probes to amplify a region of the p72 viral gene, as described previously [33]. Each sample was included in triplicates, and values were normalized to the standard positive controls. Reactions were performed using the ABI 7500 Fast real-time PCR system (Applied Biosystems, Foster City, CA, USA).

### 2.7. Western Blot Analysis

Vero cells seeded in six-well plates were infected with ASFV in the presence or absence of different concentrations of ITZ at a moi of 1 pfu/cell. Cells were lysed at different times post-infection in Laemmli sample buffer. Protein extracts were electrophoresed in 12% acrylamide-bisacrylamide gels, transferred to a nitrocellulose membrane (Bio-Rad, Hercules, CA, USA) and detected with corresponding antibodies anti-p30, anti-p72 and tubulin. As a secondary antibody, anti-mouse IgG (GE Healthcare, Chicago, IL, USA) or anti-rabbit IgG (Bio-Rad) conjugated to horseradish peroxidase was used at a 1:5000 dilution. β-Tubulin (Sigma) was used as a load control in Western blotting (WB) analysis. Finally, bands obtained after development with ECL reagent were detected on a Molecular Imager Chemidoc XRSplus imaging system. Bands were quantified by densitometry and data normalized to control values using Image lab software (Bio-Rad).

### 2.8. Flow Cytometry

Vero cells were pretreated with inhibitors at the indicated concentrations, followed by infection with Ba71V or BPP30GFV at a moi of 1 pfu/cell. At 16 hpi, cells were washed with PBS and harvested by trypsinization. Flow cytometry experiments were performed by detection of infected cells with an anti-p72 monoclonal antibody followed by incubation with FITC-conjugated anti-mouse inmmunoglubulins 1:50, diluted in FACS buffer (Dako). When using the recombinant BPP30GFP green fluorescent the use of antibodies was not necessary. 10,000 cells per tube in triplicates were scored and analyzed in a FACS Canto II flow cytometer (BD Sciences, San Diego, CA, USA) to determine the percentage of infected cells per condition. Infected cell percentages obtained after inhibitors treatment were normalized to control values.

### 2.9. Fluorescence and Confocal Microscopy

Cells were grown on glass coverslips and fixed in PBS 4% paraformaldehyde (PFA) for 15 min and permeabilized with PBS-0.1% Triton X-100 for 10 min. After blocking with BSA (Sigma), cells were incubated with corresponding antibodies, and nuclei were stained with Topro3 (Molecular Probes) before mounting.

Confocal microscopy was carried out in a Leica TCS SPE confocal microscope using a 63× immersion oil objective. Conventional fluorescence microscopy to analyze Filipin staining was performed in a Leica DM RB microscope, through a 63× immersion oil objective. Image analyses were performed with Leica Application Suite advanced fluorescence (LAS AF Lite) (Leica Microsystems, Wetzlar, Germany) and ImageJ software version 1.47v for Windows (National Institute of Health and LOCI, Wisconsin (Madison, WI, USA). Colocalization was determined through “Just Another Colocalization Plugin” plugin of ImageJ software (JACoP, https://imagej.nih.gov/ij/plugins/track/jacop.html, Institut Curie, Orsay, France).

### 2.10. Cholesterol Staining

Free intracellular cholesterol was detected using fluorescent filipin (Sigma) as previously described [34,35,36]. Filipin signal was recorded using a 390–415-nm-wavelength excitation filter and a 450–470-nm-wavelength emission filter.

### 2.11. Statistical Analyses

Statistical analyses were performed using GraphPad Prism 6 software (San Diego, CA, USA). All experiments were performed more than two times, and data are presented as the means ± the standard deviation (SD) from independent experiments. Differences between groups were assessed with the Bonferroni test. Metrics were normalized to control values. Asterisks denote statistically significant differences (****, *P* < 0.0001, ***, *P* < 0.001; **, *P* < 0.01; and *, *P* < 0.05).

## 3. Results

### 3.1. Redistribution of Endosomes and Cholesterol to ASFV Viral Factories

**Figure 1:**
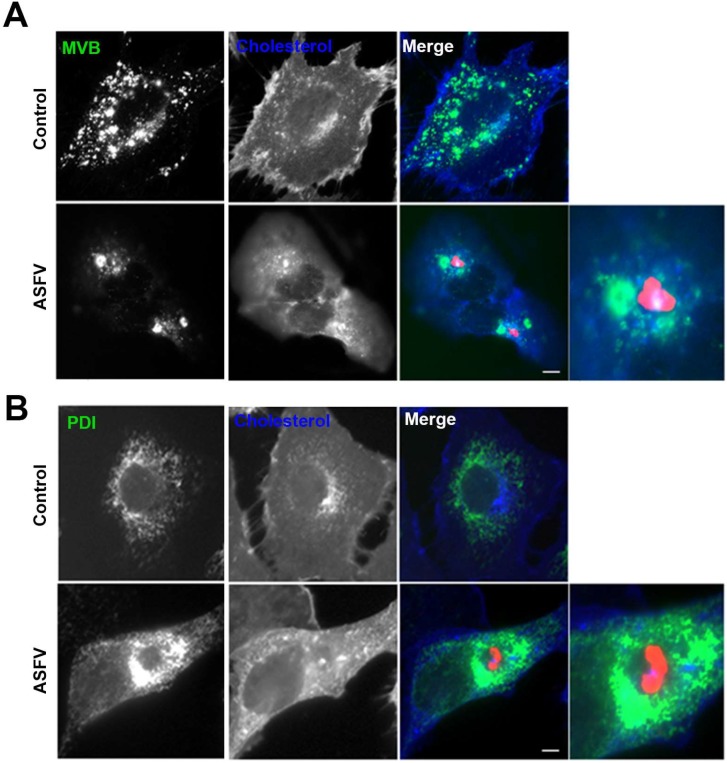
Redistribution of endosomes and cholesterol to ASFV VFs. Representative fluorescence micrographs of mock-infected (control) or infected Vero cells with ASFV for 16 h and stained for (**A**) multivesicular bodies (MVB; green) or (**B**) ER marker PDI (green) and cholesterol (blue). Infected cells were labelled for viral protein p54 concentrated at the VFs (red). (**A**) Diffuse pattern of endosomes and cholesterol in controls changed as they were recruited in clusters to the VFs in infected cells. Zoom images show details of endosomes in close proximity to areas of viral protein accumulation. (**B**) ER also redistributed around but external to the VFs leaving a void space next to the viral protein accumulation. Bar 10 μm.

Endosomal membranes are also very likely to be involved in the formation of the replication sites. Figure 1 shows the distribution of cholesterol and late endosomes called multivesicular bodies (MVB) diffusely dispersed in the cytoplasm. Endosomes store cholesterol and can redistribute this lipid from the endosomal membrane to the ER. Upon infection, the distribution of endosomes change as they get recruited to the viral replication site.

### 3.2. ITZ Disrupts Cholesterol Accumulation around Factories

**Figure 2:**
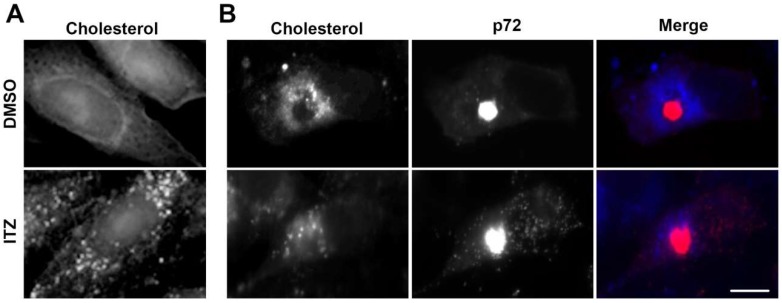
ITZ impairs cholesterol recruitment to the VFs. (**A**) Cells were treated either with ITZ or DMSO for 16 h before fixation and filipin labeling (blue). ITZ treated cells showed an altered distribution of cholesterol to punctate vesicular structures resembling endosomes. (**B**) Cells were pretreated with ITZ or DMSO and infected with ASFV for 16 h. Viral factories showed intense labelling of anti-p72 antibody. Infected cells treated with ITZ lacked the characteristic redistribution of cholesterol around the viral replication sites. Bar 10 μm.

ASFV infection is highly dependent on cholesterol. Then, we evaluated the impact of ITZ in the cholesterol distribution in Vero cells. We found that ITZ redistributed cholesterol in uninfected control cells. In contrast to the common diffuse distribution of cholesterol in control cells, in the presence of ITZ, cholesterol accumulated in vesicles resembling endosomes (Figure 2A).

ASFV remodels intracellular cholesterol and redistributes free cholesterol to viral replication sites [14]. ITZ reduced the number of cells with viral factories. However, in the few cells in which the viral factory formed, cholesterol deposition around the viral factory was reduced (Figure 2B).

### 3.3. ASFV Dependence on Cholesterol Transport Mediated by OSBP

**Figure 3:**
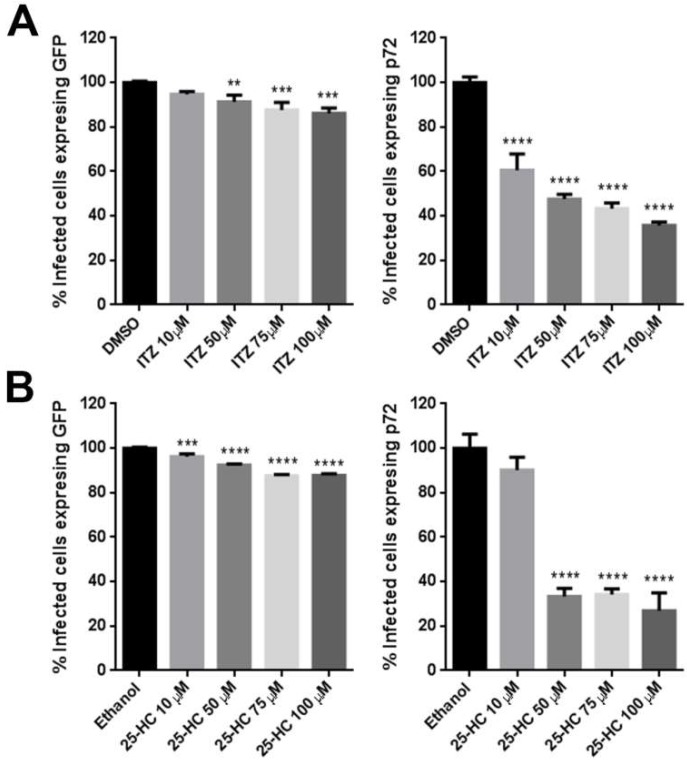
Effect of ITZ and 25-HC inhibitors on ASFV infection. Vero cells were pretreated with (**A**) ITZ or (**B**) 25-HC and infected with Ba71V or Bp30GFP for 16 hpi. Infected cells were detected by flow cytometry and percentages normalized to values in cells treated with the solvent DMSO or Ethanol. Error bars indicate S.D. from three independent experiments. Statistically significant differences are indicated by asterisks (*** *p* < 0.001, ** *p* < 0.01; * *p* < 0.05).

Then, we analyzed whether ITZ has any effect on ASFV infection, potentially interfering OSBP function in the transport of cholesterol to ASFV replication sites. The impact of this compound on infectivity was measured by the expression of GFP and p72 proteins by flow cytometry at 16 hpi. The number of cells expressing GFP, infected with recombinant virus expressing GFP fused to the promoter of the early viral p30 protein was only slightly reduced. However, the inhibitor drastically affected the expression of the late p72 protein. ITZ caused a dose-dependent reduction in the number of cells expressing late p72 protein compared to control cells, reaching ca. 65% inhibition at 100 µM concentrations. Results were compared to controls with solvent DMSO (Figure 3A).

To confirm that the inhibitory effect or ITZ on ASFV infection involved OSBP, infected cells were pretreated with 25-HC, a high-affinity ligand of OSBP that inhibits cholesterol transfer by OSBP. We found similar results as shown in Figure 3B. The addition of 25-HC significantly reduced ASFV infection ca. 70% and its effect was marked at late times of infection.

**Figure 4:**
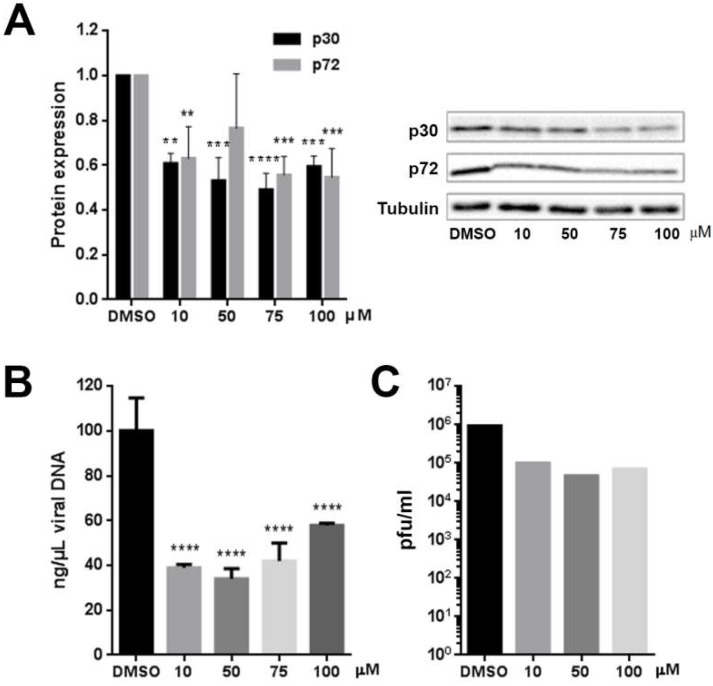
(**A**) ITZ treatment reduced viral protein expression by WB after Ba71V infection. (**B**) ASFV genome copy number reduction by PCR real time and (**C**) Effect of ITZ on virus titers by plaque assay in Ba71V infected Vero cells at 24 hpi. Error bars indicate S.D. from three independent experiments. Statistically significant differences are indicated by asterisks (**** *p* <0.0001, *** *p* < 0.001, ** *p* < 0.01).

Next, we evaluated other infection parameters. The expression of viral proteins was analyzed by Western blotting of infected Vero cells extracts and viral DNA replication was analyzed by real-time PCR, both at 16 hpi. Compared to cells treated with DMSO, the amount of viral protein production, p30 and p72 was reduced in cells treated with ITZ (Figure 4A). As expected, reductions found in other infection parameters with ITZ, correlated with a decrease in viral replication at 16 hpi (Figure 4B) and virus titers reduced ca. 1 log (Figure 4C).

### 3.4. ITZ Does Not Change OSBP Localization in Vero Cells

**Figure 5:**
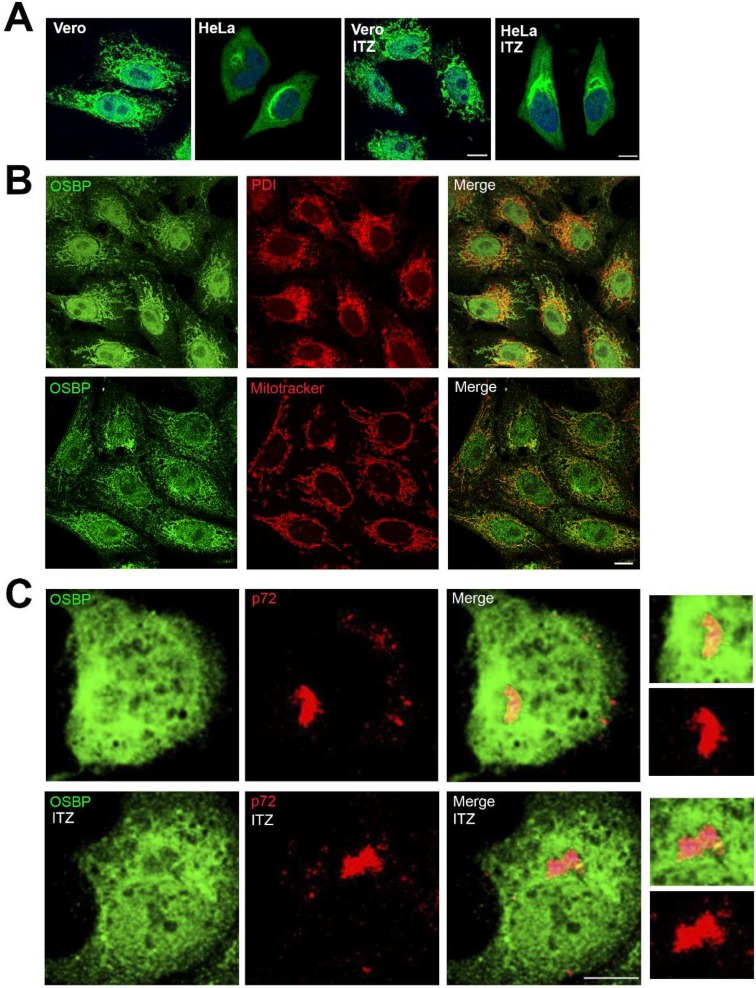
Redistribution of OSBP in ASFV infected cells independently of ITZ. (**A**) OSBP distribution in HeLa and Vero cells treated with 25 μM ITZ or DMSO for 16 h, fixed and stained with antibody against OSBP (green). DNA was stained with Topro3 (blue). Bar 10 μm. (**B**) Vero cells were stained with antibody against OSBP (green) and PDI or Mitotracker (red). Bar 10 μm. (**C**) Vero cells were treated with ITZ or DMSO, infected with ASFV for 16 h and immunostained for viral capsid protein p72 (red) and OSBP (green). Merged image depicts colocalization in yellow at the external boundaries of the VFs. Detail in the zoom images. Bar 25 μm.

ITZ inhibited the accumulation of cholesterol on VFs and these are virus-induced structures that are essential for virus replication. OSBP is involved in transport of cholesterol and PI4P between cellular membranes and it has been identified as a target for ITZ [26]. Then, we investigated whether ITZ affects cellular distribution of OSBP in Vero cells by indirect immunofluorescence. We observed a punctate-tubular pattern for OSBP staining, resembling ER or mitochondria and there were not apparent differences between cells treated with ITZ or left untreated. It had been previously described that OSBP is localized in the cytosol of Hela cells with a faintly visible Golgi pattern, while treatment with ITZ resulted in relocalization of OSBP to the Golgi complex [26]. However, we found a different distribution for OSBP in Vero compared to HeLa cells (Figure 5A).

To ascertain whether the OSBP punctate-tubular staining corresponded to the endoplasmic reticulum or mitochondria, we double labelled cells with anti-PDI antibody or a mitochondrion-selective vital dye (MitoTracker). The immunofluorescence pattern presented colocalization of OSBP with mitochondria (Pearson correlation = 0.51 ± 0.06) and only partial colocalization with ER in Vero cells (Pearson correlation = 0.2 ± 0.11; Figure 5B).

Next question was OSBP localization in infected cells. Our results showed that ASFV infection induced changes in OSBP pattern in Vero cells at 16 hpi. We found a partial co-localization with p72, a structural viral protein that accumulates at VFs at 16 hpi. OSBP redistributed in close apposition to the membrane boundaries of the VF (Figure 5C, zoom). This redistribution of OSBP appeared to be independent of ITZ treatment in ASFV-infected cells.

### 3.5. ASFV Recruits PI4Kβ and ACBD3 around VFs

**Figure 6:**
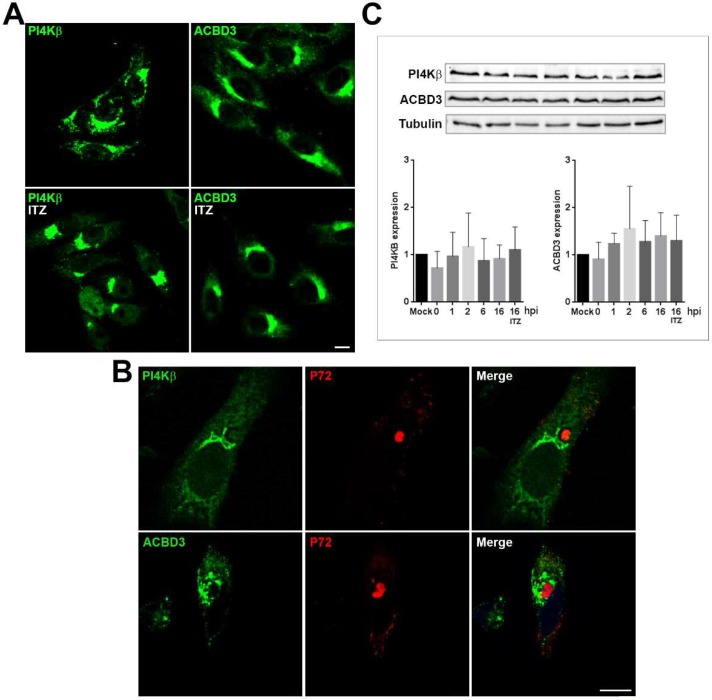
OSBP-related proteins PI4Kβ and ACBD3 redistribute to areas around VFs. (**A**) Mock-infected Vero cells treated with ITZ or DMSO or (**B**) infected with ASFV for 16 h and stained for ACBD3 or PI4Kβ (green). Infected cells were labelled for viral protein p72 (red). PI4Kβ distributed around the VF, leaving a ring devoid of staining around the replication organelle. ACBD3 was redistributed and accumulated at the VFs and presented a vesicular staining. Bar 10 μm. (**C**) Graphs show PI4Kβ and ACBD3 expression at several times postinfection (1–16 hpi) and under ITZ treatment at 16 hpi. Quantification of the bands was corrected to tubulin data and normalized to control (mock) values. A representative WB image is shown.

Next, to analyze whether ITZ had any effect on other MCSs proteins related with lipid transport, we studied localization of PI4Kβ and ACBD3 proteins in Vero cells. ACBD3 and PI4Kβ showed a Golgi-like distribution without apparent differences between ITZ treated cells or controls (Figure 6A).

Immunofluorescence analysis of PI4Kβ and ACBD3 in ASFV-infected cells showed that ASFV induced a marked redistribution of these lipid-shuttling proteins. Infected cells (Figure 6B) were stained with antibodies against PI4Kβ and ACBD3 and viral factory was stained with an antibody against p72 viral protein. PI4Kβ was mainly distributed surrounding the VF. ASFV disrupted Golgi pattern of ACBD3 and distributed it around the VF in a vesicular pattern resembling endosomal structures (Figure 6B).

We used western blot analysis to evaluate the expression levels of PI4Kβ and ACBD3 over the course of the infection at several time points in control cells with DMSO and in presence of ITZ at 16 hpi. Interestingly, ASFV did not induce significant changes on the expression these proteins. Tubulin levels remained constant during the course of the infection (Figure 6C).

## 4. Discussion

African swine fever virus (ASFV) infection remodels intracellular cholesterol by increasing its cellular uptake and redistributes free cholesterol to viral replication sites to ensure an appropriate lipid flux to establish productive infection [14]. In addition, intact cholesterol efflux from endosomes is required for ASFV release to the cytoplasm [14]. These facts indicate an important role of lipids and sterol transport for ASFV replication. In fact, little is known about the cellular factors and mechanisms underlying virus directed remodeling of host membranes into specialized membranous structures where the viral genome replicates. Recent studies have implicated MCSs machinery and sterol shuttling proteins such as oxysterol-binding protein (OSBP) and OSBP-related proteins (ORPs), in several viral replication processes [21,37]. ITZ was identified as a novel inhibitor of enteroviruses, cardioviruses, dengue virus and HCV by targeting sterol-shuttling proteins OSBP and ORP4 [26,38].

We found that ITZ treatment decreased ASFV infectivity and viral replication. ITZ barely affected GFP positive infected cells, but drastically reduced the expression of the late p72 protein. Because GFP is expressed under the control of the early protein p30 promoter, these results indicate that ITZ did not affect entry or early gene expression. Our results suggest that, similar to other viral diseases, this effect could be due to the reduction of lipid supplies to the viral replication sites as shown by reduction of VFs and cholesterol depletion around VFs.

ASFV infection causes reorganization of endosomes, in order to build a compact viral replication organelle [18]. Endosomes are the source of extracellular cholesterol that enters the endocytic pathway bound to low-density-lipoproteins (LDL) and accumulates at MVBs [39]. When cholesterol levels decrease, free cholesterol is transferred from the endosomal membrane to other cellular destinations, such as the ER. On the endosomal membrane, cholesterol mediates interaction between Rab7-interacting lysosomal protein (RILP) and the dynein-dynactin complex through OSBP-related protein 1L (ORP1L), which is known to regulate late endosome positioning [40]. Hence, cholesterol membrane content could direct endosome redistribution.

OSBP is a cytoplasmic protein with a high affinity for oxysterols. It belongs to a family of proteins characterized by a C-terminal OSBP-related ligand-binding domain (ORD) that binds oxysterols or cholesterol [41]. OSBP-related proteins (ORP) carry a pleckstrin homology (PH) domain that targets organelle membranes via phosphoinositides and two phenylalanines in an acidic tract (FFAT) motif [42]. These molecules are abundant at membrane contact sites (MCSs) providing an efficient way to transfer lipids, which do not diffuse freely between membrane compartments.

A recent study revealed that ITZ acts inhibiting enteroviruses and hepatitis C (HCV) replication by targeting oxysterol-binding protein (OSBP) and OSBP-related protein 4 (ORP4). Direct binding of ITZ to OSBP, which localizes at viral replication organelles, disrupts its lipid-shuttling function, accounting for the antiviral effect of ITZ [26].

To ascertain that OSBP is a relevant target at ASFV infection, infected cells were pretreated with 25-HC. 25-HC binds to OSBP with high affinity, acting as an inhibitor of cholesterol transfer by OSBP [29,30]. We found that 25-HC also inhibited significantly ASFV infectivity.

OSBP is a central regulator of lipid homeostasis at MCSs between the ER and other organelles. Under low cholesterol conditions or ITZ treatment, OSBP concentrates at a perinuclear region or at Golgi apparatus in HeLa cells overexpressing this protein [26,29]. Our observations suggested that OSBP display another distribution in Vero cells and localizes to mitochondria and ER. It is hardly surprising to find this shuttling protein in several cytoplasmic membranes emphasizing its dynamic function in membrane interfaces. Mitochondrial distribution could indicate that, in Vero cells, OSBP could be related to OSBP-related proteins ORP5 and ORP8. These proteins are localized at ER–mitochondria contacts and support the maintenance of mitochondria morphology and respiratory function [43]. Apparently, ASFV remodeled the mitochondrial-ER pattern of OSBP redirecting this protein to the VF as a potential indicative of the high-energy demand of the viral cycle. In fact, ASFV remodels the mitochondrial network to its own benefit [44]. OSBP was localized at the VF periphery, where viral proteins are synthesized at the ER. ASFV infection caused redistribution of OSBP around the VF, independent of ITZ treatment.

Those findings, together with the fact that cholesterol is essential for ASFV productive infection, suggest that the antiviral activity of ITZ and 25-HC, in this specific case, could be due to the disruption of cholesterol shuttling to the VFs [28], implying that ASFV is strictly dependent on these lipid supplies for replication. Consistently, we found reduced cholesterol accumulation around VFs with ITZ. However, ITZ did not affect expression or localization of other proteins related with lipid transport at MCSs such as ACBD3, PI4Kβ. Upon ASFV infection, however, PI4Kβ distributes around VFs, while ACBD3 distributes closely to the VFs in a vesicular pattern resembling endosomal structures, it is likely that as recruitment of PI4Kβ to Coxsackievirus B3 replication organelles, it is independent of ACBD3 [45].

In conclusion, these results strengthen the relevance of cholesterol, as a molecular target for ASFV. Also, OSBP could be important for lipid recruitment to VFs in ASFV infection as it is distributed at the membrane boundaries of the replication sites. Our study reveals that ITZ and 25-HC decreased ASFV replication but did not fully prevent the viral cycle progression and finally, PI4Kβ and ACBD3 were redistributed to viral factories and could be implicated in sterol shuttling required for ASFV VFs formation.
